# Latent profile and network analysis of risk perception among a sample of Chinese university students during the COVID-19 pandemic: a cross-sectional and longitudinal study

**DOI:** 10.3389/fpubh.2023.1171870

**Published:** 2024-01-05

**Authors:** Zhimin Niu, Ligang Liu, Songli Mei, Li Li

**Affiliations:** ^1^Department of Health Law, Gannan Medical University, Ganzhou, China; ^2^School of Economics and Management, Jiangxi University of Science and Technology, Ganzhou, China; ^3^School of Public Health, Jilin University, Changchun, China; ^4^School of Humanities and Social Sciences, Gannan Medical University, Ganzhou, China

**Keywords:** risk perception, latent profile analysis, network analysis, cross-sectional study, longitudinal study, Chinese university students

## Abstract

**Background:**

The risk perception of contracting COVID-19 is an important topic for assessing and predicting COVID-19 infection and health education during the pandemic. However, studies that use latent profiles and network analysis together to measure the risk perception of COVID-19 are rare. Therefore, this study combined latent profile analysis and network analysis to measure risk perception toward COVID-19 among Chinese university students through a cross-sectional and longitudinal study.

**Methods:**

A sample of 1,837 Chinese university students (735 males, 40%) completed the cross-sectional study with an eight-item risk perception questionnaire in January 2020, while 334 Chinese university students (111 males, 33.2%) completed the longitudinal study at three time points.

**Results:**

A two-class model including a low risk perception class (*n* = 1,005, 54.7%) and a high risk perception class (*n* = 832, 45.3%) was selected for the cross-sectional study. Nodes rp6 (“*Average people have chances of contracting COVID-19''*) and rp7 (“*Average people worry about catching COVID-19”*) had the strongest edge intensity (*r* = 0.491), while node rp5 (“*The COVID-19 outbreak affects the whole country”*) had the highest strength centrality in the cross-sectional study. The risk perception of contracting COVID-19 decreased continuously at the three time points. Moreover, the network structures and global strengths had no significant differences in the longitudinal study.

**Conclusions:**

The risk perception of contracting COVID-19 decreased continually during the COVID-19 pandemic, which indicated the importance of cultural influence and effective government management in China. In addition, university students displayed strong trust and confidence in the government's ability to fight COVID-19. The results indicate that the government should take strong measures to prevent and intervene in various risks and reinforce the public's trust through positive media communications.

## 1 Introduction

Risk perception is regarded as a subjective and intuitive judgement that people make, such as the risk of contracting an illness, being injured or dying (1). Risk perception also refers to “an individual's perceived susceptibility to a threat” and has three components: deliberative, affective, and experience (2). In addition, risk perception may be classified into two dimensions: cognition and emotion from a psychological perspective or individual and public from a demographic perspective (1, 3). High perceived probability and strong feelings of anxiety or nervousness may jointly correspond to high risk perception (4). Moreover, behavior motivation theory and risk reappraisal theory propose a reciprocal relationship between risk perception and protective behaviors (5). Risk analysts have indicated that there is a significant gender difference in risk perception in different situations (6), which may originate from gender structure, especially gendered ideology and gendered practice. For example, women generally worry about health and safety issues, which causes them to perceive high environmental risk (7).

Coronavirus disease 2019 (COVID-19), described as a contagious disease by the World Health Organization (8), spread quickly from January 2020 and still influences public health worldwide. Some scholars reported that perceived COVID-19 infection risk significantly increased from March 10 to March 31, 2020 (9). Dryhurst et al. also assessed the public's risk perception of COVID-19 worldwide between March and April 2020 and reported factors influencing risk perception toward COVID-19, such as prosocial values, individual knowledge and protective health behaviors (10). Individuals' awareness of COVID-19 risk may influence preventative health behaviors and increase or reduce the negative outcome of COVID-19. Risk perception of COVID-19 as a social phenomenon may contribute to managing public health risks. A longitudinal study from March 2020 to January 2021 investigated the stability of the psychological factors (e.g., prosociality, trust, and efficacy) of risk perception toward COVID-19 and found that psychological factors may predict better risk perception of COVID-19 (11).

When COVID-19 broke out in 2019, the disease was an unfamiliar risk that filled people with dread. As time went on, the risk perception of COVID-19 may have changed for different groups in different countries. A two-wave longitudinal study reported that COVID-19 risk perception declined in an analysis from March 2020 to July 2020 among an Italian population (12). Another study reported that few relationships between risk perception of COVID-19 and protective behaviors were found in a sample of the Chinese population due to conforming behaviors, while risk perception of COVID-19 and protective behaviors influenced each other in the later stage of COVID-19 in the United States (13). Health behavior theory cannot be used to explain this difference, which may be associated with culture, policy, and the stage of the COVID-19 evolution. Moreover, high risk perception of COVID-19 predicted worse psychological problems, such as depression and anxiety (14, 15). Individuals often believe that they have a lower risk of infectious diseases than others, which is regarded as optimism bias of risk perception (16). In society, social media, culture and policy may influence the public's risk perception of COVID-19.

Latent profile analysis (LPA) refers to a statistical method that focuses on identifying subpopulations or latent profiles, which requires continuous measured variables (17). LPA may offer many advantages over traditional multiple regression and cluster analysis, such as describing multiple profiles and analyzing the relationships of risk perception and other variables in the present study. Network analysis is a powerful tool to identify patterns and trends in the relationships between multiple variables to better understand the structure and function of complex systems. Network analysis is also a relatively new and promising approach for modeling interactions between many variables and is represented by a visual graph.

Although risk perception during the COVID-19 pandemic has been studied from multiple perspectives using different methods, latent profile analysis and network analysis have rarely been used together in cross-sectional and longitudinal studies. A large sample is needed for latent profile analysis. In addition, the supporting evidence for the internal structure of risk perception of contracting COVID-19 needs to be examined through a longitudinal study. Therefore, the aims of the present study were to (i) explore latent classes of risk perception toward COVID-19 among a sample of Chinese university students during the COVID-19 breakout (T1); (ii) examine gender differences in risk perception toward COVID-19 during the COVID-19 breakout (T1); and (iii) utilize a network comparison test to examine the change in risk perception toward COVID-19 in a longitudinal study (T1, T2, and T3).

## 2 Methods

### 2.1 Participants

A convenience sample of university students from four provinces of China was collected, and the self-report survey was completed through the online Wenjuanxing Platform. The cross-sectional study included 1,837 students (735 males, 40% and 1,102 females, 60%) at T1. Of the participants, 863 (47%) students lived in urban areas, and 974 (53%) lived in rural areas. In the T2 study, the 1,166 students included 431 males (38.6%) and 685 females (61.4%), 531 (47.6%) lived in urban areas, and 585 (52.4%) lived in rural areas. In the T3 study, 334 students (111 males, 33.2% and 223 females, 66.8%) completed the survey. There were 160 (47.9%) and 174 (52.1%) participants in urban and rural areas, respectively. With the time change, some students had graduated, and some refused to attend or did not complete the late surveys (T2 and T3).

The participants ranged in age from 18 to 25 years (mean = 19.0 years; SD = 1.8 years).

### 2.2 Procedures

The study was conducted from January 2020 to September 2021 at three time points (T1: January 2020, T2: January 2021, T3: September 2021). In January 2020, the first survey was conducted in five universities in four provinces of China (i.e., Jiangxi, Heilongjiang, Shannxi and Liaoning). University teachers invited students to take the online survey voluntarily. To facilitate the longitudinal study, all participants were asked to report their exclusive identity numbers. Among them, 835 participants submitted the second survey in January 2021. Next, 529 participants submitted the third survey in July 2021. Some respondents returned the third survey with incomplete or questionable answers (i.e., the respondents answered the same options) and were excluded from further participation, leaving 334 participants for the longitudinal study.

### 2.3 Ethics

Ethical approval was obtained from the research team's university, and oral informed consent was received from participants who were advised about the aim of the study and their ability to withdraw at any time.

### 2.4 Measures

The risk perception questionnaire originated from a study by Yan and Wen (3) and has four dimensions: individual risk perception, public risk perception, individuals' behaviors and interpersonal communication. In the present study, eight items were selected, including two dimensions: individual risk perception and public risk perception. The dimension of individuals' risk perception included four items: “*The COVID-19 outbreak is closely related to me*,” “*I have chances of contracting COVID-19*,” “*I worry about catching COVID-19*,” and “*I think the COVID-19 outbreak is serious*,” while the public's risk perception included another four items: “*The COVID-19 outbreak affects the whole country*,” “*Average people have chances of contracting COVID-19*,” “*Average people worry about catching COVID-19*,” and “*Everyone thinks that the COVID-19 outbreak is serious*” (Appendix 1). Participants answered using a 5-point Likert scale ranging from 1 (“totally disagree”) to 5 (“totally agree”). Higher total scores represent higher levels of risk perception. The Cronbach's alpha and McDonald's ω of the risk perception questionnaire were 0.807 and 0.805, 0.66 and 0.69 for individual risk perception, and 0.79 and 0.81 for public risk perception, respectively.

### 2.5 Statistical analysis

Likert scales are primarily used in many social science fields because they capture the level of agreement or respondents' feelings about a specific topic. Although the variables of interest derived from Likert scales are measured on ordinal scales, when the sample size is large enough, researchers typically apply parametric tests for statistical hypothesis testing due to the underlying asymptotic results emphasizing the normal distribution.

Descriptive statistics, the reliability of the risk perception questionnaire, and network analysis were conducted using Jeffrey's Amazing Statistics Program (JASP) version 0.16.1.0 (18). Latent profile analysis was performed using Mplus version 9. In addition, the network comparison test (NCT) was conducted utilizing R version 4.2.2 (19).

Normal data distribution was described through skewness, kurtosis (20, 21), and quantile-quantile plot (QQ-plot). JASP reduced the kurtosis formula by 3 to compare the resulting parameter with the value zero. The gender difference in risk perception toward COVID-19 was assessed using two-sided independent *t-*tests.

Latent profile analysis (LPA) was performed to identify and describe the optimal number of profiles for risk perception toward COVID-19 among a sample of Chinese university students. Two to five profiles were conducted for all participants. The optimal number of profiles was based on the concept of risk perception toward COVID-19, smallest estimated class proportions (should be more than 5% of the total sample), and statistical model fit indices including the Akaike information criteria (AIC), Bayesian information criterion (BIC), sample size-adjusted BIC (A-BIC), Lo-Mendell-Rubin-adjusted likelihood ratio test (LMRA-LRT), and bootstrap likelihood ratio test (BLRT) (22–25). The model fit indices, including decreased AIC, BIC, A-BIC and the LMRA-LRT and BLRT with a significant *p*-value (<0.01), may indicate a better model fit (26). Replication analysis was conducted for cross-validation through two split samples at random (*n*1 = 892, *n*2 = 945). Moreover, multinomial logistic regression was conducted with gender and residential status as covariates and risk perception classes as the outcome variable (i.e., high risk perception class as the reference class), and *t*-tests were performed to examine the difference between classes.

Network analysis is an effective and visual method of studying the interaction between multiple variables or the structure of some variables through nodes (i.e., variables) and edges (i.e., the connection of variables). The network model was assessed through the graphic least absolute shrinkage and selection operator (LASSO) method, which originated from the Extended Bayesian Information Criterion (i.e., EBICglasso) (27, 28). The indices, which included betweenness, closeness, strength and expected influence, represented the centrality of nodes (29, 30). In addition, the network accuracy was examined through edge-weight accuracy, centrality stability and testing for significant differences in nodes and edges (28). Non-parametric bootstrapping (i.e., 1,000 samples) was utilized to calculate edge-weight accuracy and test for significant differences in nodes and edges, while case-dropping subset bootstrapping (95% confidence intervals) was utilized to assess the stability of centrality indices (28). The correlation stability coefficient (*CS*-coefficient, at least ≥0.25) indicated node centrality stability (28). The strong and weak connections of nodes were indicated by thick edges and thin edges, respectively. The blue edge and orange edge represent positive and negative correlations between variables, respectively. The network comparison test (NCT) was conducted across time (T1, T2, and T3).

Latent profile analysis (LPA) was used to identify latent subpopulations with perceived risk of contracting COVID-19 based on a sample of Chinese university students, while network analysis examined the interaction of dimensions of risk perception.

## 3 Results

### 3.1 Descriptive statistics of risk perception in the cross-sectional study (T1)

For skewness (<2) and kurtosis (<7), based on previous studies (20, 21), most items were considered normally distributed, except item 5 (skewness = −2.218, kurtosis = 7.973). The QQ plot also showed that the data were approximately normal (Appendix 2). The corrected item-total correlation of eight items ranged from 0.43 to 0.66. Alpha if item deleted (eight items: 0.77–0.80) and factor loading (eight items: 0.39–0.68) indicated that the risk perception questionnaire had fitted psychometric characteristics (Appendices 1, 2). In addition, there was no significant gender difference in risk perception among the 1,837 participants (all Cohen's *d* < 0.2, Appendix 3).

### 3.2 Latent profile analysis of risk perception toward COVID-19 in the cross-sectional study (T1)

**Figure 1:**
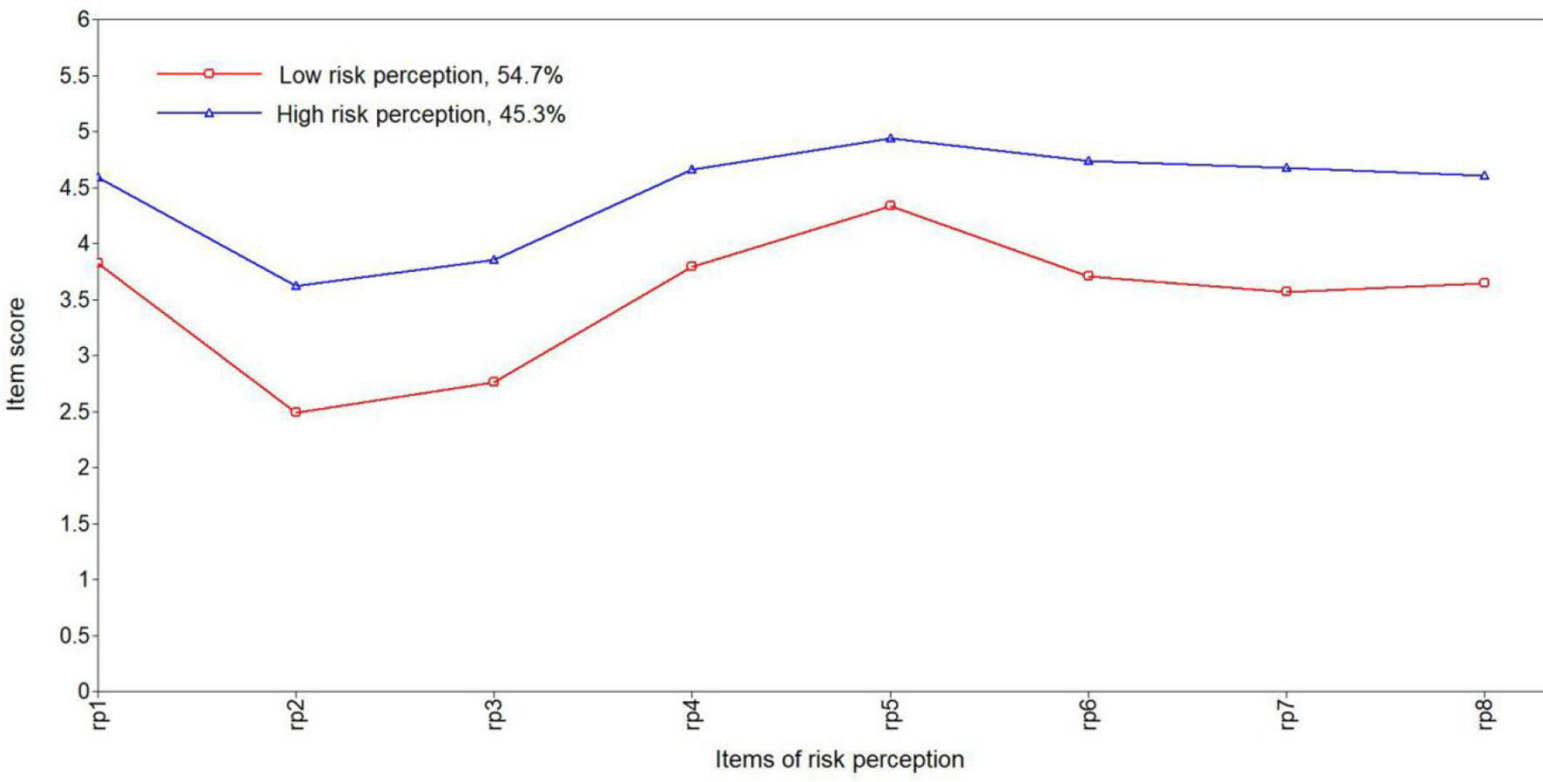
Latent class profile of risk perception toward COVID-19.

The fit indices and class membership size of LPA are shown in Table 1. The two-class model including the low risk perception of the COVID-19 class (*n* = 1,005, 54.7%) and the high risk perception of the COVID-19 class (*n* = 832, 45.3%) was selected as the optimal model based on the fit indices and interpretability of the model (Figure 1). The three-class, four-class and five-class models had decreasing values of AIC, BIC, A-BIC, and good entropy; however, only the two-class model had a significant value of LMR-LTR (<0.001). In addition, the results of the replication analysis also indicated that the two-class model had a more suitable class membership size than the three-class model (<5% of the total sample). The high posterior probabilities of memberships of the two latent classes were 0.929 and 0.944, respectively. Multinomial logistic regression was performed with gender and residential status as covariates. There were no class differences between males and females (χ^2^ = 0.26, *p* = 0.61, Phi = −0.012) or between those living in urban and rural areas (χ^2^ = 0.351, *p* = 0.553, Phi = −0.014; Appendices 4, 5). The *t-*tests of two factors, eight items and total score of risk perception between the two classes were examined, and significant differences were found between the two classes (all *p* < 0.001 and Cohen's *d* > 0.8; Appendix 6).

**Table 1:** Fit indices for LPA of eight items on risk perception toward COVID-19 among 1,837 participants.

	**Model**	***k***	**AIC**	**BIC**	**A-BIC**	**Entropy**	**LMR-LTR *(p*)**	**BLRT *(p*)**	**Class membership size**
Total sample	Class 2	25	37,070.02	37,207.92	37,128.49	0.78	**< 0.001**	< 0.001	1,005 (54.7%)/832(45.3%)
	Class 3	34	35,787.53	35,975.07	35,867.05	0.88	0.26	< 0.001	120 (6.5%)/1,043 (56.8%)/674 (36.7)
	Class 4	43	32,011.16	32,248.34	32,111.73	1.00	0.14	< 0.001	**50** (2.7%)/799 (43.5%)/272 (14.8%)/716 (39%)
	Class 5	52	31,724.12	32,010.94	31,845.74	0.98	0.10	< 0.001	**50** (2.7%)/106 (5.8%)/799 (43.5%)/166 (9%)/716 (39%)
Sample 1	Class 2	25	17,609.86	17,729.70	17,650.30	0.80	**< 0.001**	< 0.001	487 (54.6%)/405 (45.4%)
Sample 2	Class 2	25	19,455.84	19,577.12	19,497.72	0.76	**< 0.01**	< 0.001	536 (56.7%)/409 (43.3%)
Sample 1	Class 3	34	16,969.30	17,132.28	17,024.30	0.90	0.02	< 0.001	63 (7.1%)/335 (37.5%)/494 (55.4%)
Sample 2	Class 3	34	18,786.07	18,951.01	18,843.03	0.88	0.34	< 0.001	**14** (1.5%)/558 (59%)/373 (39.5%)

AIC, Akaike Information Criterion; BIC, Bayesian Information Criterion; A-BIC, Sample Size-Adjusted BIC; LMR-LRT, LoMendell Rubin Likelihood Ratio Test; BLRT, Bootstrap Likelihood Ratio Test. The LMR-LTR with a significant value (< 0.01) and smallest estimated class proportions should be more than 5% of the total sample.

### 3.3 Network analysis of risk perception toward COVID-19 in the cross-sectional study (T1)

**Figure 2:**
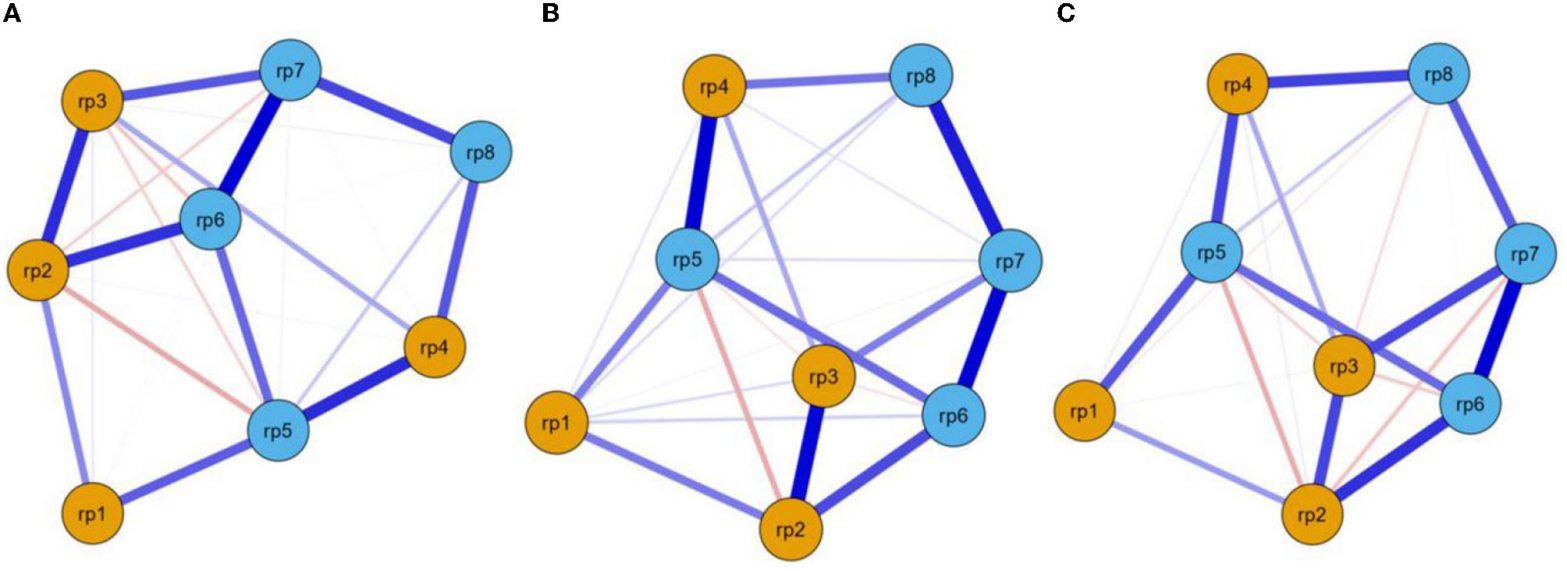
EBICglasso model based on network analysis according to risk perception among all participants **(A)**, males **(B)**, and females **(C)**. rp–rp4 = individual's risk perception, rp5–rp8 = the public's risk perception.

The EBICglasso networks of eight-item risk perception toward COVID-19, including all participants (*n* = 1,837), males (*n* = 735), and females (*n* = 1,102), are shown in Figures 2A–C. For the network of all participants, rp6 (“*Average people have chances of contracting COVID-19*”) and rp7 (“*Average people worry about catching COVID-19*”) had the strongest edge intensity (*r* = 0.491; Appendix 7). Node rp5 (“*The COVID-19 outbreak affects the whole country*”) had the highest strength centrality (0.999, Appendix 8). The edge-weight accuracy and centrality stability are shown in Appendix 9. The narrow gray area indicates that the bootstrapped CIs may be appropriate to interpret the edge-weight accuracy. The stability of node strength (CS > 0.5) represented better centrality stability. The tests for significant differences indicated that the edges rp6 (“*Average people have chances of contracting COVID-19*”)—rp7 (“*Average people worry about catching COVID-19*”) and rp2 (“*I have chances of contracting COVID-19*”)—rp3 (“*I worry about catching COVID-19*”) were significantly different from each other. All node strengths were also significantly different from each other. In the different gender networks, rp4 (“*I think the COVID-19 outbreak is serious*”) and rp5 (“*The COVID-19 outbreak affects the whole country*”) (*r* = 0.436), and rp6–rp7 (*r* = 0.516) were the strongest edges among males and females, respectively. The node rp5 was the strongest node for males (strength = 1.338) and females (strength = 0.895; Appendices 10–12).

### 3.4 Risk perception of COVID-19 in the longitudinal study (T1–T3)

**Figure 3:**
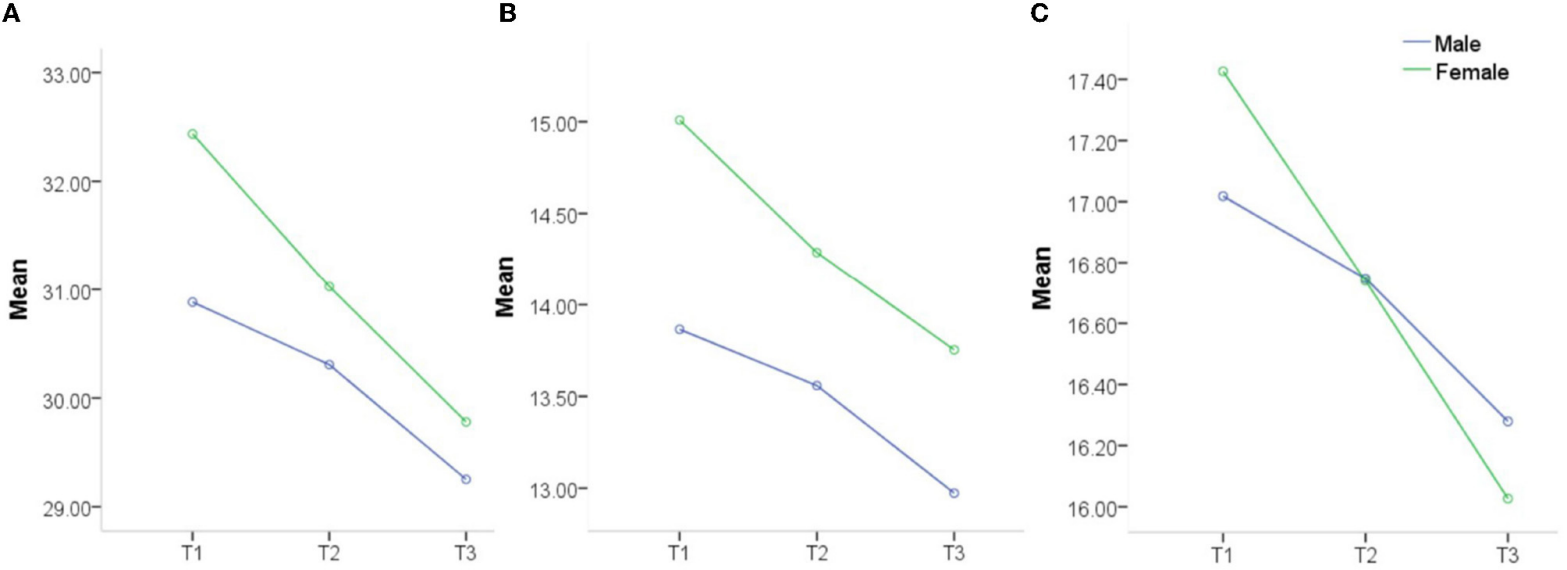
Risk perception of COVID-19 between genders at three time points (T1, T2, and T3). **(A)** Total risk perception score, **(B)** Individual risk perception score, and **(C)** Public risk perception score.

The risk perception of COVID-19 significantly differed among the three time points (η^2^ > 0.01; Appendix 13). The risk perception of COVID-19 decreased continuously at the three time points for all participants (Figure 3). In addition, the items rp1 (“*The COVID-19 outbreak is closely related to me*”) and rp2 (“*I have chances of contracting COVID-19*”) had gender differences at the three time points (all *p* < 0.05 and Cohen's *d* > 0.2; Appendix 14). However, a significant correlation was not found between the total score of risk perception at the three time points (T1, T2, and T3) and gender [log(BF_10_) < 3; Appendix 15].

### 3.5 Network comparison of risk perception of COVID-19 in the longitudinal study (T1–T3)

**Figure 4:**
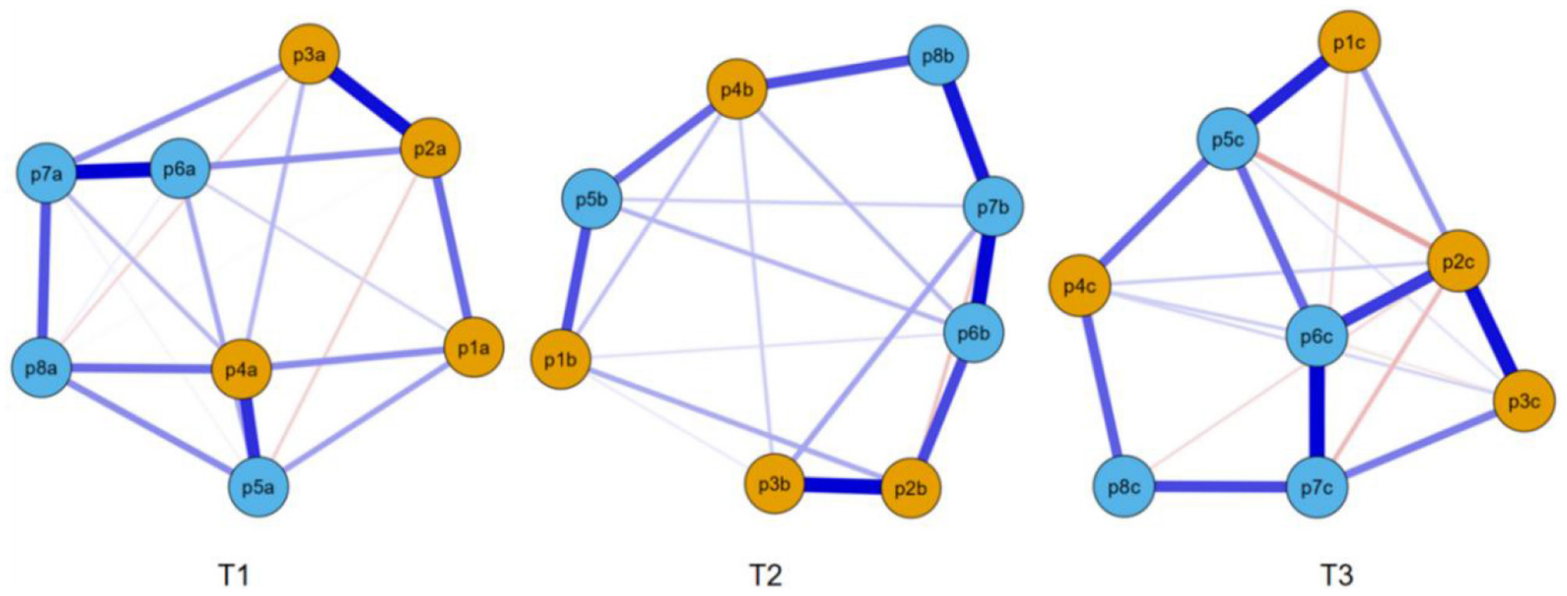
EBICglasso model based on network analysis according to risk perception at three different times. rp1–rp4 = individual risk perception, rp5–rp8 = public risk perception.

The EBICglasso networks of risk perception at the three time points are shown in Figure 4 (T1, T2, and T3). Nodes rp6 (“*Average people have chances of contracting COVID-19*”) and rp7 (“*Average people worry about catching COVID-19*”) had the strongest edge intensity at TI (*r* = 0.464) and T3 (*r* = 0.471), while nodes rp2 (“*I have chances of contracting COVID-19*”) and rp3 (“*I worry about catching COVID-19*”) had the strongest edge intensity at TI (*r* = 0.423; Appendices 16–18). Node rp7 had the highest strength centrality at TI (1.266) and T2 (1.648), while node rp2 (1.432) had the highest strength centrality at T3 (Appendix 19).

The network structures had no significant differences between T1 and T2 (M = 0.175, *p* = 0.252), between T1 and T3 (M = 0.213, *p* = 0.064), or between T2 and T3 (M = 0.159, *p* = 0.397). In addition, the global strengths also had no significant differences between T1 and T2 (3.27 vs. 3.06, *p* = 0.261), between T1 and T3 (3.27 vs. 2.74, *p* = 0.075), or between T2 and T3 (3.06 vs. 2.74, *p* = 0.402).

## 4 Discussion

The questionnaire about risk perception toward COVID-19 had good psychometric characteristics, which was similar to the results of Yan and Wen (3). Although some scholars have reported that gender influenced risk perception toward COVID-19 among the Pakistani population, gender differences were only shown in the “trust government” dimension, not in other dimensions (i.e., fear, attitude, awareness, all *p* > 0.05) (31). Another study indicated that females had higher risk perception toward COVID-19 than males, but the effect of COVID-19 was the same in different individuals' lives (32). In addition, a survey from the United States also reported that females had higher danger perception and fear of COVID-19 (33). In the present study, a gender difference in risk perception toward COVID-19 was not found, which was consistent with Li et al.'s study (13). The possible reason is that the Chinese government took many strong measures to increase the public's trust and confidence through media broadcasts about health education and interventions, travel restrictions and social distancing at the beginning of the COVID-19 pandemic and effectively provided COVID-19 vaccinations. Moreover, China is a typical collectivist country in which the public commonly trusts, supports and advocates for the government. Therefore, cultural differences, strong policies, and the positive effects of social media may influence the risk perception of COVID-19 among the Chinese population, including university students.

A survey reported four classes, including low-, mild-, moderate-, and high-risk perceptions of COVID-19, among Chinese nurse clinicians (34). Another study identified three classes, risk neutral, risk deniers, and risk exaggerators, among the Chinese population during the COVID-19 pandemic (35). Replication analysis as a method of verifying LPA was not conducted in these studies. In addition, Kleitman et al. (36) classified two groups, including the compliant group and non-compliant group, for protective behaviors during the COVID-19 outbreak. In the compliant group, people perceived a high risk of contracting COVID-19, and in the non-compliant group, members perceived a low risk. In the present study, the low entropy value of the two-class model may be related to the large sample size, which was not considered the important fit index of LPA due to the poor statistical capacity (37). In addition, the two-class (i.e., high and low) model of risk perception toward COVID-19 may be explained and distinguished more easily by average people, which is also suitable for individuals of different genders and living statuses. Due to the lack of a validity assessment tool, the receiver operating characteristic curve (ROC) and the threshold value were not available in the present study.

The cross-sectional results of network analysis indicated that the items rp6 (“*Average people have chances of contracting COVID-19*”) and rp7 (“*Average people worry about catching COVID-19*”) were the very important components of risk perception of COVID-19, which represented the public's perception of COVID-19 risk in the questionnaire and indicated the importance of public interest in collectivistic countries. As the pillar of society and national development, Chinese university students thought “*the COVID-19 outbreak affects the whole country*” during the COVID-19 pandemic and believed that only interdependent communities and stable countries could fight the disease. Therefore, all types of rules (e.g., washing hands, wearing masks in public places, and home quarantine) were strictly observed by most people, including university students in China. Some studies also found that collectivistic regions were more likely to wear masks as cultures and countries required (38). In addition, high coping efficacy is more easily stimulated due to sufficient health information and psychological support through the collectivism system in China (39).

In the present study, the risk perception of COVID-19 significantly decreased at the three time points. The most likely reason is that the infection and mortality of COVID-19 declined rapidly from February 14, 2020 (5,090 local confirmed cases and 121 deaths), to October 31, 2021 (33 cases imported from abroad, 59 local confirmed cases, and no deaths), based on statistical data from the China National Health Commission (40, 41). By October 31, 2021, 2,274,072 doses of the COVID-19 vaccine had been given in mainland China (42), which may have decreased the risk perception of COVID-19 and increased health awareness and confidence. In mainland China, almost all university students except individuals with physical reasons received the COVID-19 vaccine based on the requirements of universities and the government.

A survey in the United States reported that COVID-19 infection and mortality may have increased the willingness to wear a mask among youth, while different political ideologies, regardless of similar risk perceptions toward COVID-19, may have influenced protective behaviors (e.g., social distancing and wearing masks) (43). In addition, the items rp1 (“*The COVID-19 outbreak is closely related to me*”) and rp2 (“*I have chances of contracting COVID-19*”) had gender differences at the three time points, but no significant correlation was found between risk perceptions and gender. A survey reported that females were more likely to take precautionary measures and reduce COVID-19 risk perception during the COVID-19 pandemic (31). However, many factors, including the role of social media, perceived understanding, coping strategies, social communication needs in real life, and trust attitudes toward the country and government, may have interacted and influenced the risk perception of COVID-19, which may explain the gender difference in risk perception (44–46).

The network structures and global strengths had no significant differences among the three time points through pairwise comparison, which may explain the stability of the risk perception structure. High risk perception of COVID-19 is more likely to trigger negative emotions (e.g., worry, anxiety, fear, and even depression) and lead to a higher level of vaccination intention for preventing and fighting against COVID-19 (47). In China, with widespread mass vaccination, declining infection and mortality, and positive precautionary measures, the risk perception of COVID-19 has continually decreased, especially for females.

From a psychological perspective, the declining risk perception of COVID-19 may be explained by psychological immunization theory, that is, repeated exposure is able to reinforce resistance to stressful events (48). Individuals at risk may more easily change their cognition and attitudes toward COVID-19 and not change their location based on cognitive dissonance theory (39, 49). In addition, protection motivation theory stresses the importance of coping efficacy, that is, beliefs and behaviors about effective responses to avoid the COVID-19 threat (39, 50). Based on media system dependency theory (51), an individual's attitudes and behaviors may be changed or reinforced through the media's information dissemination. Moreover, risk perception is socially constructed based on cultural cognition theory (52). People living in collectivistic societies are more likely to perceive COVID-19 risk and adhere to social standards than people living in individualistic cultures (12). In the present study, gender theory cannot be used to explain the risk perception of COVID-19, which indicated that cultural and policy factors may significantly influence risk perception among individuals more than gender.

Some limitations should be considered when viewing these results. First, this convenience sampling of university students may not represent all Chinese university students. Second, a single risk perception questionnaire was conducted, which led to the receiver operating characteristic curve (ROC) and the threshold value of the risk perception questionnaire being unavailable due to a lack of validity assessment tools. Third, individuals self-reported their risk perception toward COVID-19, which may be inadequate and lack objectivity. Moreover, the effect factors of risk perception toward COVID-19 and prediction of health protective behaviors may be examined together through the network analysis method.

## 5 Conclusion

Risk perception of COVID-19 declined continually during the COVID-19 pandemic in China, which indicated the importance of cultural influence and effective government management. In addition, university students displayed strong trust and confidence in the government's ability to fight COVID-19. The results may provide a reference for coping with other great risks in the future; that is, the government should take strong measures to prevent and intervene in various risks and reinforce the public's trust and confidence through positive media broadcasts.

## Data availability statement

The original contributions presented in the study are included in the article/Supplementary material, further inquiries can be directed to the corresponding author.

## Ethics statement

The studies involving humans were approved by Gannan Medical University Ethics Committee. The studies were conducted in accordance with the local legislation and institutional requirements. The participants provided their written informed consent to participate in this study.

## Author contributions

ZN and LLiu conceived and designed the experiments and wrote the first draft of the paper. ZN, LLi, and SM performed the experiments. ZN and LLi analyzed and interpreted the data. SM contributed reagents, materials, and analysis tools. All authors contributed to the article and approved the submitted version.
