# Supplementary material for: Latent profile and network analysis of risk perception among a sample of Chinese university students during the COVID-19 pandemic: a cross-sectional and longitudinal study
Source: Front Public Health. 2024 Jan 5;11:1171870. doi: 10.3389/fpubh.2023.1171870 (PMC10796724; doi:10.3389/fpubh.2023.1171870)
Supplement: Supplementary file 1 [file Table_1.DOCX]

**Appendix**

**Appendix S1**. Skewness, kurtosis, mean scores (SD), corrected item-total correlation, and internal consistency (Cronbach’s α) coefficients for items in the risk perception questionnaire at T1 (N = 1837)

|  | Item | Skewness | Kurtosis | Item M±SD | Corrected Item-total  correlation | Alpha if item deleted |
| --- | --- | --- | --- | --- | --- | --- |
| 1 | The COVID-19 outbreak is closely related to me. | -1.506 | 1.926 | 4.17±1.06 | 0.43 | 0.80 |
| 2 | I have chances of contracting COVID-19. | -0.149 | -1.104 | 3.00±1.34 | 0.52 | 0.79 |
| 3 | I worry about catching COVID-19. | -0.387 | -0.936 | 3.25±1.27 | 0.52 | 0.79 |
| 4 | I think the COVID-19 outbreak is serious. | -1.311 | 2.467 | 4.18±0.84 | 0.54 | 0.78 |
| 5 | The COVID-19 outbreak affects the whole country. | -2.218 | 7.973 | 4.61±0.63 | 0.48 | 0.79 |
| 6 | Average people have chances of contracting COVID-19. | -1.113 | 1.828 | 4.17±0.84 | 0.66 | 0.77 |
| 7 | Average people worry about catching COVID-19. | -0.958 | 1.333 | 4.07±0.85 | 0.66 | 0.77 |
| 8 | Everyone thinks that the COVID-19 outbreak is serious. | -0.799 | 0.738 | 4.08±0.84 | 0.53 | 0.79 |

**
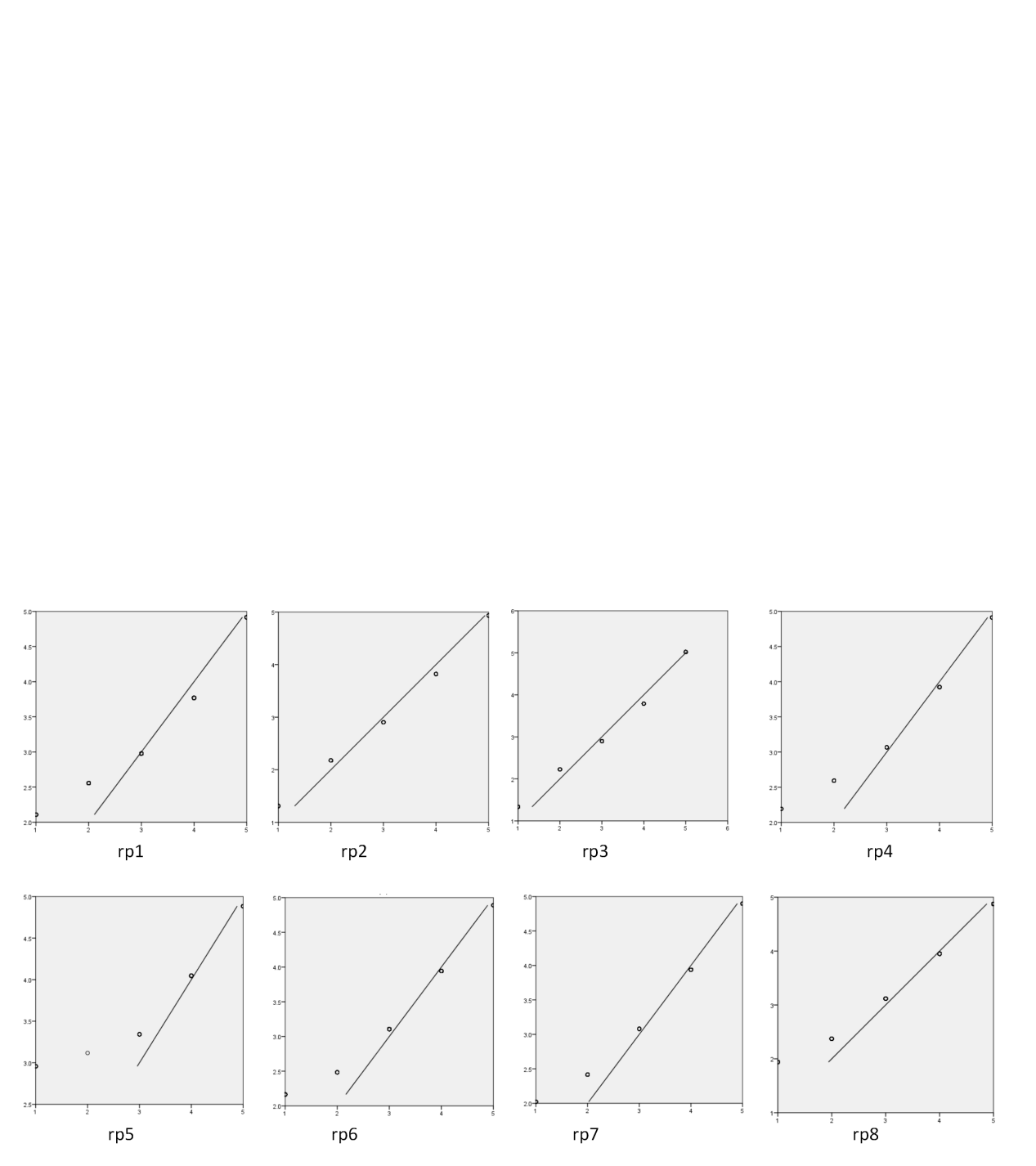
**

**Appendix S2**. QQ-plot assessing Normal data distribution for items in the risk perception questionnaire at T1 (N = 1837), X-axis: observed value, Y-axis: expected normal.

**Appendix S3**. Factor loading and gender difference of risk perception at T1

|  | Item | F1 | F2 | Male (n=735) | Female (n=1102) | *t* | *p* | *Cohen’s d* |
| --- | --- | --- | --- | --- | --- | --- | --- | --- |
| 1 | The COVID-19 outbreak is closely related to me. | 0.39 |  | 4.10±1.14 | 4.22±1.00 | 2.32 | 0.02 | 0.11 |
| 2 | I have chances of contracting COVID-19. | 0.56 |  | 2.95±1.39 | 3.04±1.30 | 1.33 | 0.19 | 0.06 |
| 3 | I worry about catching COVID-19. | 0.55 |  | 3.22±1.35 | 3.28±1.22 | 0.94 | 0.35 | 0.05 |
| 4 | I think the COVID-19 outbreak is serious. | 0.44 |  | 4.22±0.84 | 4.16±0.84 | 1.68 | 0.09 | 0.08 |
| 5 | The COVID-19 outbreak affects the whole country. |  | 0.56 | 4.59±0.66 | 4.62±0.61 | 0.88 | 0.38 | 0.04 |
| 6 | Average people have chances of contracting COVID-19. |  | 0.65 | 4.16±0.87 | 4.18±0.82 | 0.38 | 0.71 | 0.02 |
| 7 | Average people worry about catching COVID-19. |  | 0.68 | 4.07±0.87 | 4.07±0.84 | 0.20 | 0.84 | 0.01 |
| 8 | Everyone thinks that the COVID-19 outbreak is serious. |  | 0.61 | 4.08±0.89 | 4.08±0.81 | 0.09 | 0.93 | 0.01 |
|  | The COVID-19 outbreak is closely related to me. |  |  | 31.41±5.51 | 31.63±4.88 | 0.90 | 0.37 | 0.04 |

**Appendix S4**. Unstandardized multinominal logistic regression coefficients, standard errors, and odds ratios for covariates in predicting latent profile class membership, using the high risk perception class as the reference class at T1.

| Covariate | Probability of higher covariate scores for high risk perception class compared to low risk perception class | | | | | |
| --- | --- | --- | --- | --- | --- | --- |
|  | χ^2^ | B | SE | *p* | Phi | OR (95%CI) |
| Gender | 0.260 | -0.079 | 0.104 | 0.610 | 0.012 | 0.924 (0.754 – 1.131) |
| Residential status | 0.351 | -0.057 | 0.095 | 0.553 | 0.014 | 0.945 (0.785 – 1.137) |


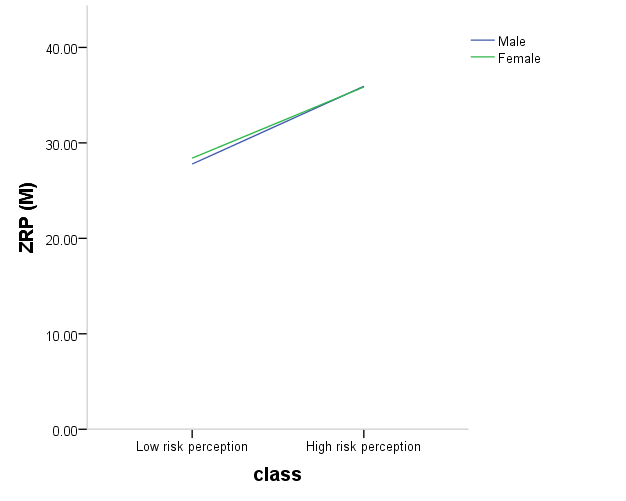


**Appendix S5** Mean plots of risk perception in two classes at T1

**Appendix S6** *T*-test of risk perception of COVID-19 between two classes at T1

|  | Low risk perception Class | | High risk perception Class | | *t* | *p* | Cohen’s d | 95% CI | |
| --- | --- | --- | --- | --- | --- | --- | --- | --- | --- |
|  | M | SD | M | SD |  |  |  |  |  |
| rp1 | 3.82 | 1.10 | 4.61 | 0.80 | 17.13 | <0.001 | 0.81 | 0.70 | 0.88 |
| rp2 | 2.51 | 1.16 | 3.64 | 1.28 | 19.77 | <0.001 | 0.93 | 1.02 | 1.24 |
| rp3 | 2.78 | 1.15 | 3.87 | 1.16 | 20.00 | <0.001 | 0.94 | 0.98 | 1.19 |
| rp4 | 3.79 | 0.83 | 4.68 | 0.54 | 26.36 | <0.001 | 1.24 | 0.82 | 0.96 |
| rp5 | 4.33 | 0.71 | 4.97 | 0.16 | 25.40 | <0.001 | 1.19 | 0.60 | 0.70 |
| rp6 | 3.70 | 0.76 | 4.77 | 0.47 | 35.17 | <0.001 | 1.65 | 1.01 | 1.13 |
| rp7 | 3.57 | 0.74 | 4.71 | 0.49 | 37.59 | <0.001 | 1.77 | 1.08 | 1.19 |
| rp8 | 3.65 | 0.75 | 4.63 | 0.59 | 30.64 | <0.001 | 1.44 | 0.92 | 1.05 |
| F1 | 12.91 | 2.66 | 16.80 | 2.46 | 32.14 | <0.001 | 1.51 | 3.66 | 4.13 |
| F2 | 15.25 | 1.92 | 19.09 | 1.08 | 50.87 | <0.001 | 2.39 | 3.69 | 3.99 |
| RP | 28.15 | 3.82 | 35.89 | 2.82 | 48.09 | <0.001 | 2.26 | 7.42 | 8.05 |

F1 = individual’s risk perception (rp1-rp4), F2 = the public’s risk perception (rp5-rp8), RP = total score of risk reception (rp1-rp8).

**Appendix S7**. Edge weight matrix of the network of risk perception among 1837 participants at T1

| Variable | rp1 | rp2 | rp3 | rp4 | rp5 | rp6 | rp7 | rp8 |
| --- | --- | --- | --- | --- | --- | --- | --- | --- |
| rp1 | 0.000 | 0.225 | 0.043 | 0.026 | 0.309 | 0.010 | 0.010 | 0.000 |
| rp2 | 0.225 | 0.000 | 0.406 | 0.016 | -0.165 | 0.395 | -0.086 | -0.010 |
| rp3 | 0.043 | **0.406** | 0.000 | 0.165 | -0.082 | -0.105 | 0.321 | -0.028 |
| rp4 | 0.026 | 0.016 | 0.165 | 0.000 | 0.406 | 0.000 | 0.012 | 0.323 |
| rp5 | 0.309 | -0.165 | -0.082 | 0.406 | 0.000 | 0.289 | 0.023 | 0.100 |
| rp6 | 0.010 | 0.395 | -0.105 | 0.000 | 0.289 | 0.000 | 0.491 | 0.000 |
| rp7 | 0.010 | -0.086 | 0.321 | 0.012 | 0.023 | **0.491** | 0.000 | 0.356 |
| rp8 | 0.000 | -0.010 | -0.028 | 0.323 | 0.100 | 0.000 | 0.356 | 0.000 |

**Appendix S8.** Centrality measures of the network of risk perception among 1837 participants at T1

| Variable | Betweenness | Closeness | Strength | Expected influence |
| --- | --- | --- | --- | --- |
| rp1 | -1.056 | -1.699 | -1.743 | -1.338 |
| rp2 | 0.096 | 0.074 | 0.740 | -0.451 |
| rp3 | -1.056 | -0.419 | 0.182 | -0.797 |
| rp4 | -0.288 | -0.346 | -0.556 | 0.478 |
| rp5 | 1.248 | 0.385 | **0.999** | 0.103 |
| rp6 | 1.632 | 1.592 | 0.691 | 1.216 |
| rp7 | 0.096 | 0.929 | 0.723 | 1.476 |
| rp8 | -0.672 | -0.515 | -1.037 | -0.688 |


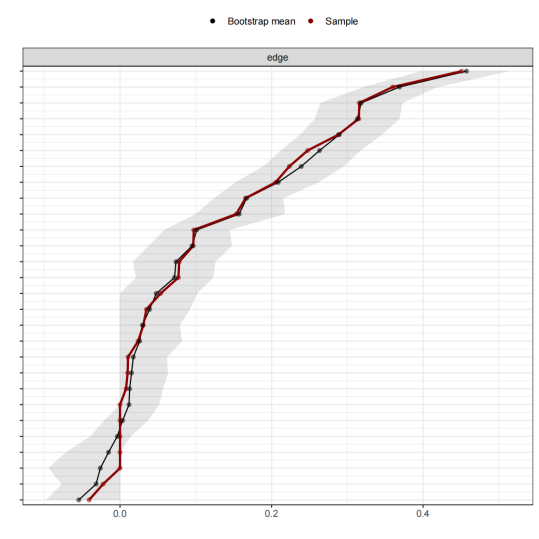
A
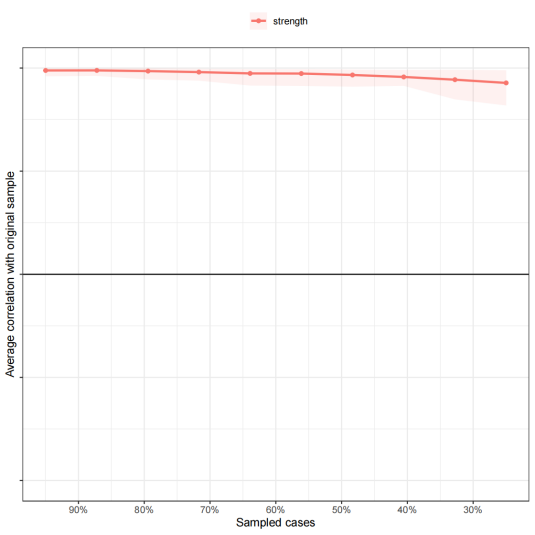
B


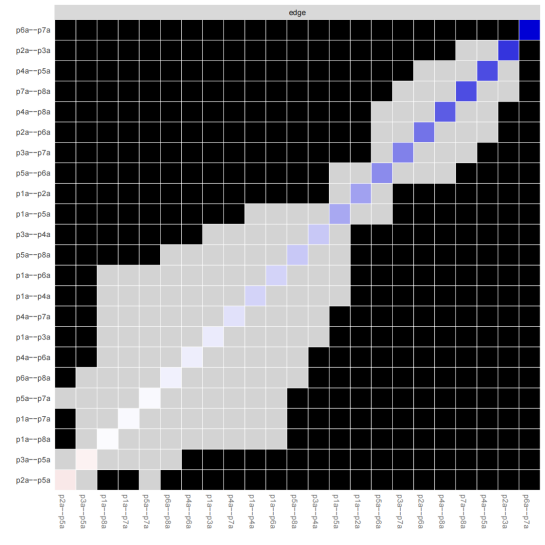
C
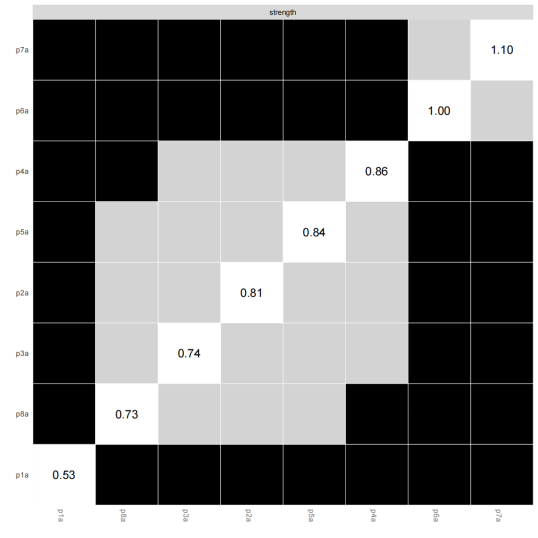
D

**Appendix S9.** Bootstrapped confidence intervals of estimated edge-weights (A) and Case-dropping bootstrap procedure for node strength (B), Bootstrapped difference tests (α = 0.05) between edge-weights that were non-zero in the estimated network (C) and node strength of the items (D).

**Appendix S10**. Edge weight matrix of the network of risk perception between gender at T1

| Variable | Males | | | | | | | | Females | | | | | | | |
| --- | --- | --- | --- | --- | --- | --- | --- | --- | --- | --- | --- | --- | --- | --- | --- | --- |
|  | rp1 | rp2 | rp3 | rp4 | rp5 | rp6 | rp7 | rp8 | rp1 | rp2 | rp3 | rp4 | rp5 | rp6 | rp7 | rp8 |
| rp1 | 0.000 | 0.225 | 0.061 | 0.042 | 0.220 | 0.078 | 0.017 | 0.058 | 0.000 | 0.202 | 0.020 | 0.035 | 0.341 | 0.000 | 0.000 | -0.038 |
| rp2 | 0.225 | 0.000 | 0.424 | 0.000 | -0.142 | 0.308 | 0.000 | 0.000 | 0.202 | 0.000 | 0.374 | 0.034 | -0.162 | 0.416 | -0.117 | -0.013 |
| rp3 | 0.061 | **0.424** | 0.000 | 0.144 | -0.047 | -0.046 | 0.215 | 0.000 | 0.020 | 0.374 | 0.000 | 0.170 | -0.086 | -0.100 | 0.368 | -0.066 |
| rp4 | 0.042 | 0.000 | 0.144 | 0.000 | **0.436** | 0.000 | 0.043 | 0.238 | 0.035 | 0.034 | 0.170 | 0.000 | 0.375 | 0.000 | 0.000 | 0.382 |
| rp5 | 0.220 | -0.142 | -0.047 | 0.436 | 0.000 | 0.252 | 0.058 | 0.093 | 0.341 | -0.162 | -0.086 | 0.375 | 0.000 | 0.300 | 0.002 | 0.107 |
| rp6 | 0.078 | 0.308 | -0.046 | 0.000 | 0.252 | 0.000 | **0.429** | 0.000 | 0.000 | 0.416 | -0.100 | 0.000 | 0.300 | 0.000 | 0.516 | 0.005 |
| rp7 | 0.017 | 0.000 | 0.215 | 0.043 | 0.058 | 0.429 | 0.000 | 0.388 | 0.000 | -0.117 | 0.368 | 0.000 | 0.002 | **0.516** | 0.000 | 0.330 |
| rp8 | 0.058 | 0.000 | 0.000 | 0.238 | 0.093 | 0.000 | 0.388 | 0.000 | -0.038 | -0.013 | -0.066 | 0.382 | 0.107 | 0.005 | 0.330 | 0.000 |

**Appendix S11.** Centrality measures of the network of risk perception between gender at T1

| Variables | Males | | | | Females | | | |
| --- | --- | --- | --- | --- | --- | --- | --- | --- |
|  | Betweenness | Closeness | Strength | Expected influence | Betweenness | Closeness | Strength | Expected influence |
| rp1 | -1.577 | -1.653 | -1.508 | -1.151 | -0.977 | -1.509 | -1.927 | -1.362 |
| rp2 | 1.079 | 0.227 | 0.560 | -0.394 | -0.621 | -0.205 | 0.680 | -0.541 |
| rp3 | -0.913 | -0.348 | -0.281 | -0.822 | -0.977 | -0.631 | 0.169 | -0.804 |
| rp4 | 0.415 | -0.281 | -0.457 | 0.199 | -0.266 | -0.365 | -0.549 | 0.698 |
| rp5 | 0.415 | 0.279 | **1.338** | -0.023 | 1.154 | 0.314 | **0.895** | 0.135 |
| rp6 | 1.079 | 1.765 | 0.642 | 0.992 | 1.509 | 1.624 | 0.754 | 1.359 |
| rp7 | 0.415 | 0.648 | 0.823 | 1.844 | 0.799 | 1.133 | 0.738 | 1.185 |
| rp8 | -0.913 | -0.635 | -1.115 | -0.646 | -0.621 | -0.360 | -0.760 | -0.670 |


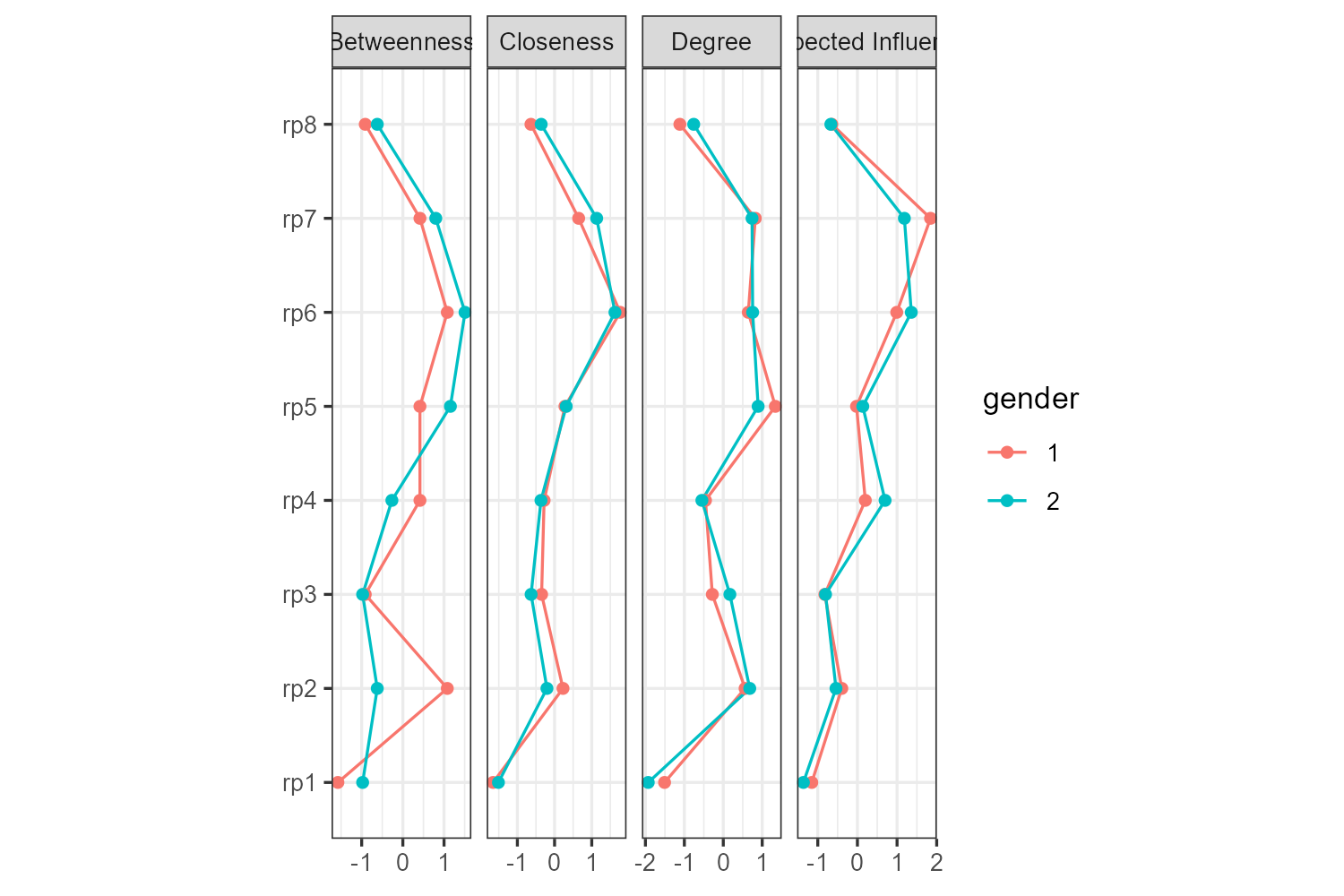


**Appendix S12.** Standardized estimates of node centrality in the network of risk perception between males (1) and females (2) at T1.

**Appendix S13.** Multiple comparison (multivariate test) of items/total score of risk perception at three time points

| Item | T1 | T2 | T3 | F | *p* | *η*^2^ |
| --- | --- | --- | --- | --- | --- | --- |
| rp1 | 4.16±1.03 | 4.14±1.00 | 4.23±0.93 | 126.922 | <0.001 | 0.434 |
| rp2 | 2.98±1.35 | 2.87±1.23 | 2.73±1.24 | 6.768 | 0.001 | 0.039 |
| rp3 | 3.25±1.30 | 3.03±1.23 | 2.87±1.27 | 15.555 | <0.001 | 0.086 |
| rp4 | 4.24±0.84 | 4.00±0.88 | 3.67±1.04 | 30.676 | <0.001 | 0.156 |
| rp5 | 4.65±0.60 | 4.69±0.5 | 4.66±0.67 | 1.992 | 0.138 | 0.012 |
| rp6 | 4.28±0.81 | 4.16±0.80 | 4.03±1.00 | 11.547 | <0.001 | 0.065 |
| rp7 | 4.17±0.84 | 3.98±0.84 | 3.83±0.95 | 18.613 | <0.001 | 0.101 |
| rp8 | 4.19±0.82 | 3.91±0.79 | 3.59±0.91 | 40.204 | <0.001 | 0.195 |
| Risk perception | 31.92±5.24 | 30.79±1.45 | 29.60±4.90 | 28.628 | <0.001 | 0.147 |
| Individual’s risk perception | 14.63±3.39 | 14.04±3.01 | 13.49±3.06 | 20.899 | <0.001 | 0.112 |
| The public’s risk perception | 17.29±2.44 | 16.74±2.14 | 16.11±2.54 | 40.874 | <0.001 | 0.198 |

Note: T1: January 2020, T2: January 2021, T3: September 2021; the effect size ofη^2^: 0.04 is small, 0.25 is medial, 0.64 is big (Ferguson,2009).

Ferguson C J. Is psychological research really as good as medical research? Effect size comparisons between psychology and medicine. Review of General Psychology, 2009, 13(2): 130-136.

**Appendix S14.** *T*-test of gender difference at three time points

| Item/Total score | Male (n=111) | Female (n=223) | *t* | *p* | *Cohen’s d* |
| --- | --- | --- | --- | --- | --- |
| rp1 (T1) | 3.97±1.21 | 4.25±0.91 | 2.313 | 0.021* | 0.269 |
| rp2 (T1) | 2.67±1.40 | 3.13±1.30 | 2.993 | 0.003* | 0.348 |
| rp3 (T1) | 3.06±1.42 | 3.35±1.22 | 1.912 | 0.057 | 0.222 |
| rp4 (T1) | 4.16±0.87 | 4.28±0.82 | 1.239 | 0.216 | 0.144 |
| rp5 (T1) | 4.62±0.59 | 4.66±0.60 | 0.608 | 0.544 | 0.071 |
| rp6 (T1) | 4.17±0.88 | 4.33±0.76 | 1.718 | 0.087 | 0.200 |
| rp7 (T1) | 4.14±0.85 | 4.18±0.84 | 0.405 | 0.686 | 0.047 |
| rp8 (T1) | 4.08±0.87 | 4.25±0.80 | 1.736 | 0.083 | 0.202 |
| rp1 (T2) | 3.96±1.19 | 4.23±0.88 | 2.340 | 0.020* | 0.272 |
| rp2 (T2) | 2.68±1.20 | 2.96±1.23 | 2.036 | 0.043* | 0.236 |
| rp3 (T2) | 2.90±1.28 | 3.09±1.20 | 1.360 | 0.175 | 0.158 |
| rp4 (T2) | 4.02±0.91 | 4.00±0.87 | 0.219 | 0.827 | 0.025 |
| rp5 (T2) | 4.63±0.60 | 4.73±0.51 | 1.517 | 0.130 | 0.176 |
| rp6 (T2) | 4.12±0.78 | 4.18±0.82 | 0.714 | 0.476 | 0.083 |
| rp7 (T2) | 4.02±0.87 | 3.96±0.82 | 0.645 | 0.519 | 0.075 |
| rp8 (T2) | 3.98±0.82 | 3.87±0.77 | 1.174 | 0.241 | 0.136 |
| rp1 (T3) | 4.01±1.11 | 4.34±0.81 | 3.104 | 0.002* | 0.361 |
| rp2 (T3) | 2.51±1.26 | 2.83±1.21 | 2.243 | 0.026* | 0.261 |
| rp3 (T3) | 2.71±1.32 | 2.94±1.24 | 1.560 | 0.120 | 0.181 |
| rp4 (T3) | 3.74±1.07 | 3.64±1.03 | 0.842 | 0.400 | 0.098 |
| rp5 (T3) | 4.61±0.70 | 4.68±0.65 | 0.894 | 0.372 | 0.104 |
| rp6 (T3) | 3.99±1.01 | 4.05±0.99 | 0.504 | 0.614 | 0.059 |
| rp7 (T3) | 3.89±0.95 | 3.80±0.96 | 0.845 | 0.399 | 0.098 |
| rp8 (T3) | 3.78±0.99 | 3.50±0.86 | 2.728 | 0.007* | 0.317 |
| Risk perception (T1) | 30.88±5.33 | 32.44±5.13 | 2.570 | 0.011* | 0.298 |
| Risk perception (T2) | 30.31±4.46 | 31.03±4.44 | 1.395 | 0.164 | 0.162 |
| Risk perception (T3) | 29.25±5.12 | 29.78±4.79 | 0.928 | 0.354 | 0.108 |
| Individual’s risk perception (T1) | 13.86±3.57 | 15.01±3.24 | 2.937 | 0.004* | 0.341 |
| Individual’s risk perception (T2) | 13.56±3.02 | 14.29±2.98 | 2.093 | 0.037* | 0.243 |
| Individual’s risk perception (T3) | 12.97±3.08 | 13.75±3.02 | 2.210 | 0.028* | 0.257 |
| The public’s risk perception (T1) | 17.02±2.46 | 17.43±2.43 | 1.440 | 0.151 | 0.167 |
| The public’s risk perception (T2) | 16.75±2.29 | 16.74±2.07 | 0.031 | 0.975 | 0.004 |
| The public’s risk perception (T3) | 16.28±2.66 | 16.03±2.48 | 0.855 | 0.393 | 0.099 |

Note: T1: January 2020, T2: January 2021, T3: September 2021

**Appendix S15.** Correlation between Risk perception at three time points and gender

|  | T1 | T2 | T3 |
| --- | --- | --- | --- |
| T2 | 0.635 |  |  |
| log(BF_10_) | 82.381 |  |  |
| T3 | 0.487 | 0.765 |  |
| log(BF_10_) | 41.947 | 143.362 |  |
| Gender | 0.140 | 0.076 | 0.051 |
| log(BF_10_) | **0.569** | 1.717 | 2.253 |

Note: T1: January 2020, T2: January 2021, T3: September 2021

**Appendix S16**. Edge weight matrix of the network of risk perception among 334 participants at T1

| Variable | rp1 | rp2 | rp3 | rp4 | rp5 | rp6 | rp7 | rp8 |
| --- | --- | --- | --- | --- | --- | --- | --- | --- |
| rp1 | 0.000 | 0.267 | 0.000 | 0.206 | 0.169 | 0.080 | 0.000 | 0.000 |
| rp2 | 0.267 | 0.000 | 0.435 | 0.000 | -0.081 | 0.207 | 0.000 | -0.007 |
| rp3 | 0.000 | **0.435** | 0.000 | 0.129 | 0.000 | 0.000 | 0.205 | -0.069 |
| rp4 | 0.206 | 0.000 | 0.129 | 0.000 | 0.378 | 0.000 | 0.124 | 0.266 |
| rp5 | 0.169 | -0.081 | 0.000 | 0.378 | 0.000 | 0.157 | 0.020 | 0.206 |
| rp6 | 0.080 | 0.207 | 0.000 | 0.000 | 0.157 | 0.000 | 0.464 | 0.027 |
| rp7 | 0.000 | 0.000 | 0.205 | 0.124 | 0.020 | **0.464** | 0.000 | 0.311 |
| rp8 | 0.000 | -0.007 | -0.069 | 0.266 | 0.206 | 0.027 | 0.311 | 0.000 |

**Appendix S17**. Edge weight matrix of the network of risk perception among 334 participants at T2

| Variable | rp1 | rp2 | rp3 | rp4 | rp5 | rp6 | rp7 | rp8 |
| --- | --- | --- | --- | --- | --- | --- | --- | --- |
| rp1 | 0.000 | 0.137 | 0.035 | 0.099 | 0.291 | 0.052 | 0.000 | 0.000 |
| rp2 | 0.137 | 0.000 | 0.423 | 0.000 | 0.000 | 0.300 | -0.095 | 0.000 |
| rp3 | 0.035 | **0.423** | 0.000 | 0.082 | 0.000 | 0.000 | 0.148 | 0.000 |
| rp4 | 0.099 | 0.000 | 0.082 | 0.000 | 0.252 | 0.110 | 4.051e-4 | 0.292 |
| rp5 | 0.291 | 0.000 | 0.000 | 0.252 | 0.000 | 0.117 | 0.082 | 0.000 |
| rp6 | 0.052 | 0.300 | 0.000 | 0.110 | 0.117 | 0.000 | **0.416** | 0.000 |
| rp7 | 0.000 | -0.095 | 0.148 | 4.051e-4 | 0.082 | 0.416 | 0.000 | 0.387 |
| rp8 | 0.000 | 0.000 | 0.000 | 0.292 | 0.000 | 0.000 | 0.387 | 0.000 |

**Appendix S18**. Edge weight matrix of the network of risk perception among 334 participants at T3

| Variable | rp1 | rp2 | rp3 | rp4 | rp5 | rp6 | rp7 | rp8 |
| --- | --- | --- | --- | --- | --- | --- | --- | --- |
| rp1 | 0.000 | 0.185 | 0.000 | 0.000 | 0.405 | 0.010 | -0.071 | 0.000 |
| rp2 | 0.185 | 0.000 | 0.444 | 0.090 | -0.166 | 0.361 | -0.122 | -0.065 |
| rp3 | 0.000 | **0.444** | 0.000 | 0.077 | 0.052 | -0.047 | 0.239 | 0.000 |
| rp4 | 0.000 | 0.090 | 0.077 | 0.000 | 0.276 | 0.088 | 0.000 | 0.296 |
| rp5 | 0.405 | -0.166 | 0.052 | 0.276 | 0.000 | 0.275 | 0.000 | 0.000 |
| rp6 | 0.010 | 0.361 | -0.047 | 0.088 | 0.275 | 0.000 | **0.471** | 0.000 |
| rp7 | -0.071 | -0.122 | 0.239 | 0.000 | 0.000 | 0.471 | 0.000 | 0.336 |
| rp8 | 0.000 | -0.065 | 0.000 | 0.296 | 0.000 | 0.000 | 0.336 | 0.000 |

**Appendix S19.** Centrality measures of the network of risk perception at three time points

| Variables | T1 | | | | T2 | | | | T3 | | | |
| --- | --- | --- | --- | --- | --- | --- | --- | --- | --- | --- | --- | --- |
|  | Betweenness | Closeness | Strength | Expected influence | Betweenness | Closeness | Strength | Expected influence | Betweenness | Closeness | Strength | Expected influence |
| rp1 | -0.100 | -1.414 | -1.697 | -0.909 | -0.651 | -1.359 | -1.193 | -1.274 | -1.271 | -0.889 | -1.206 | -1.310 |
| rp2 | 0.702 | -0.568 | 0.337 | -0.312 | 1.086 | 0.309 | 0.694 | -0.127 | 0.182 | 0.136 | **1.432** | -0.293 |
| rp3 | -1.705 | -0.509 | -0.844 | -1.040 | -1.810 | -0.982 | -0.782 | -0.711 | -1.271 | -0.844 | -0.555 | -0.099 |
| rp4 | -0.100 | 0.920 | 1.116 | 1.377 | -0.072 | -0.365 | 0.033 | 0.404 | -0.303 | -1.073 | -0.663 | 0.227 |
| rp5 | -0.903 | -1.079 | 0.438 | -0.143 | -0.651 | -0.728 | -0.483 | -0.302 | 1.634 | 0.535 | 0.536 | 0.301 |
| rp6 | 0.702 | 0.991 | -0.129 | 0.365 | 0.507 | 1.379 | 0.916 | 1.612 | 0.666 | 1.645 | 0.805 | 1.922 |
| rp7 | 1.504 | 0.991 | **1.266** | 1.499 | 1.086 | 0.814 | **1.648** | 1.179 | 0.666 | 1.024 | 0.762 | 0.358 |
| rp8 | -0.100 | 0.667 | -0.487 | -0.836 | 0.507 | 0.931 | -0.833 | -0.781 | -0.303 | -0.535 | -1.110 | -1.107 |
